# The Influence of Orthodontic Treatment on Periodontal Health between Challenge and Synergy: A Narrative Review

**DOI:** 10.3390/dj12040112

**Published:** 2024-04-17

**Authors:** Ionut Luchian, Zenovia Surlari, Ancuta Goriuc, Nicoleta Ioanid, Irina Zetu, Oana Butnaru, Monica-Mihaela Scutariu, Monica Tatarciuc, Dana-Gabriela Budala

**Affiliations:** 1 Department of Periodontology, Faculty of Dental Medicine, “Grigore T. Popa” University of Medicine and Pharmacy, 16 Universității Street, 700115 Iasi, Romania; ionut.luchian@umfiasi.ro; 2 Department of Prosthodontics, Faculty of Dental Medicine, “Grigore T. Popa” University of Medicine and Pharmacy, 16 Universității Street, 700115 Iasi, Romania; zenovia.surlari@umfiasi.ro (Z.S.); dana-gabriela.bosinceanu@umfiasi.ro (D.-G.B.); 3 Department of Biochemistry, Faculty of Dental Medicine, “Grigore T. Popa” University of Medicine and Pharmacy, 16 Universității Street, 700115 Iasi, Romania; 4 Department of Orthodontics, Faculty of Dental Medicine, “Grigore T. Popa” University of Medicine and Pharmacy, 16 Universității Street, 700115 Iasi, Romania; irina.zetu@umfiasi.ro (I.Z.); oana.maria.butnaru@umfiasi.ro (O.B.); 5 Department of Oro-Dental Diagnosis, Faculty of Dental Medicine, “Grigore T. Popa” University of Medicine and Pharmacy, 16 Universității Street, 700115 Iasi, Romania; monica.scutariu@umfiasi.ro; 6 Department of Dental Technology, Faculty of Dental Medicine, “Grigore T. Popa” University of Medicine and Pharmacy, 16 Universității Street, 700115 Iasi, Romania; monica.tatarciuc@umfiasi.ro

**Keywords:** periodontal health, periodontal disease, periodontitis, fixed appliances, orthodontic treatment, removable appliances, aligners

## Abstract

By correctly repositioning teeth, orthodontic therapy improves both the function and appearance of an occlusion. The relationship between teeth and the tissues that surround and support them significantly influences these alterations. With ever more adults seeking orthodontic care, orthodontists are increasingly seeing patients with periodontal issues. Concerns about the patient’s appearance, such as uneven gingival margins or functional issues caused by inflammatory periodontal diseases, should be accounted for when designing orthodontic treatment plans. Furthermore, orthodontics may increase the chances of saving and recovering a degraded dentition in cases of severe periodontitis. Today, general dentists, dontists, and orthodontists play integrative roles that enable them to achieve the best possible results for their patients. This review will improve the results of interdisciplinary treatments and increase cooperation between dental specialists by drawing attention to the essential connection between orthodontics and periodontics in regular clinical practice.

## 1. Introduction

Orthodontic treatment ensures the accurate positioning of the teeth and optimizes the occlusion–jaw relationship. This approach not only improves the quality of life by helping patients with eating, talking, and their appearance [1] but also improves their overall health. Therefore, the number of adult patients choosing orthodontic treatment has steadily risen in recent years.

Effective collaboration, coordination, and communication between various dental specialists are crucial for ensuring more accurate diagnosis and optimized treatment planning. Interdisciplinary interaction is paramount and, in certain instances, facilitates coordinated dental therapy [2].

The effect of orthodontic treatment on the prevalence of periodontitis has been debated among scholars. Recently, periodontal health’s importance has increased in line with the number of adult orthodontic patients. The orthodontic treatment–periodontitis relationship has been widely studied by scholars, and it often has a synergistic character. Orthodontic treatments enhance periodontal health by aligning teeth and balancing occlusion, in turn improving hygiene by making it easier for patients to access their teeth and reducing occlusal trauma. Fixed orthodontic devices can enhance supragingival biofilm formation and worsen periodontal tissue’s condition [2]. Orthodontic pressures can lead to inflammation in the periodontium. This reaction is essential for the orthodontic tooth movement process. One challenge in orthodontics is performing treatment without impacting the root and periodontium [2].

Orthodontic tooth movement has enhanced periodontal health in numerous cases, while periodontal therapy frequently aids the promotion of orthodontic tooth movement [3].

This process could cause potential complications in dentitions impacted by chronic periodontitis. Adult patients are challenging for orthodontists to treat due to their elevated aesthetic expectations and existing oral problems potentially impeding treatment, such as tooth wear, inadequate restorations, and periodontal disease [4]. Adults afflicted with periodontal disease may undergo supplementary orthodontic therapy before prosthetic rehabilitation, whether to improve the aesthetics of their smile or address functional issues. This orthodontic treatment typically forms part of a multidisciplinary treatment plan. Nevertheless, a lack of adequate data exists regarding the advantages and disadvantages of using comprehensive orthodontic treatment to treat this specific patient population [2].

Also, scholars have disagreed about the best times and methods for periodontal therapies. Most patients suffering from periodontal disease are best suited to non-surgical periodontal therapy methods that decrease microbial populations [2]. Osseous or pocket reduction/elimination surgery, which are types of periodontal therapy, should not be performed until the end of orthodontic treatment because shifting teeth might alter the shape of the gums and bone [3,4].

Several factors, including host resistance, systemic diseases/conditions such as diabetes mellitus or a smoking habit, the periodontal phenotype (particularly for the width of the buccal bone plate), the quantity and type of dental plaque, and the patient’s oral hygiene routine are all important for maintaining periodontal health during orthodontic treatment [5,6].

Bone levels and shapes in periodontally affected patients can benefit from orthodontic therapy as it facilitates plaque removal, a reduction in occlusal trauma, and potential action to stimulate bone growth within bony defects [7,8]. On the other hand, periodontal therapy might improve the efficiency of orthodontic treatment. Scholars often advise that practitioners perform orthodontic treatment after periodontal therapy to prevent quick and irreparable damage inflammation-related gum damage during such treatment.

Orthodontic treatment, like any other form of medicine, has both positive and negative aspects. Nonetheless, compared to other surgical and non-surgical procedures, this treatment’s reported risk and complications are quite low. By highlighting the crucial relationship between orthodontics and periodontics in everyday clinical practice, we aimed to enhance multidisciplinary treatments’ success and foster more collaboration between dental experts.

The current narrative review’s objective was to establish new clinical perspectives on the ortho-periodontal synergistic relationship to improve these conditions’ prognosis.

In this study, we used the following keywords: periodontal health, periodontal disease, periodontitis, fixed appliances, orthodontic treatment, removable appliances, and aligners; two specialists investigated these keywords independently using the PubMed and Web of Science databases. To avoid the risk of bias, the investigators included both positive and negative papers on this topic. Only the publications selected by both independent investigators were considered reliable and included in this narrative review. As a result of this process, 119 publications were selected.

## 2. Mechanisms of Action

The orthodontic and periodontal professions have a common objective, namely improving both the face’s appearance and dental aesthetics while maintaining the masticatory apparatus’ health and lifespan. In recent decades, a consistent rise in the number of adult patients seeking orthodontic treatment with fixed equipment has been noted. Furthermore, a recent survey revealed that 50% of 30-year-old individuals in the United States exhibit periodontitis symptoms. This correlation between periodontal and orthodontic therapies, particularly in adult patients, has encouraged clinicians and academics to explore numerous related lines of inquiry [9].

The propagation of orthodontic forces within the strained tissue matrix to the adjacent cells in the periodontal ligament and alveolar bone leads to these cells releasing pro-inflammatory, angiogenic, and osteogenic substances, initiating the process of remodeling the periodontal ligament and alveolar bone [10]. According to the literature, fixed orthodontic appliances (FA) are associated with notable clinical attachment loss and alter the subgingival bacterial microbiota and gingival inflammation, regardless of the individual’s dental hygiene practices [11].

The presence of orthodontic brackets and elastic modules hinders the efficient elimination of plaque, elevating the individual’s susceptibility to gingivitis. Self-ligating brackets’ (SLBs) purported advantages compared to conventional brackets (CB) include improved bacterial retention and reduced plaque buildup. Past research has indicated that orthodontic treatment, if not well managed, potentially impacts the inflammatory process and causes the periodontium to deteriorate, leading to a significant loss of attachment [12].

Although the prevailing belief is that SLBs lead to enhanced oral hygiene compared to CB, several studies have challenged this notion. The debate around this topic remains unresolved. Potential alternatives to fixed orthodontic appliances (FA) include utilizing transparent aligners. These aligners have several possible benefits, such as reduced plaque buildup and enhanced gingival and periodontal parameters, which may be more advantageous than the use of FA [13].

One past study clarified the correlation between orthodontic movement and periodontal disease based on their combined impact on tissue degradation by increasing the concentrations of pro-inflammatory cytokines [14].

The way in which the jaws and gums react to orthodontic treatment is largely determined based on the duration and severity of the forces applied to the teeth. The bioelectric and pressure–tension theories can be used to assess the orthodontic biological mechanisms.

Our first hypothesis states that bending the bone produces piezoelectric currents, in turn determining changes in bone metabolism. Orthodontic stresses usually cause alveolar bone displacement, and these strains alter the periodontal ligament, formed by electrons traveling from side to side within the network of a crystalline material.

**Figure 1:**
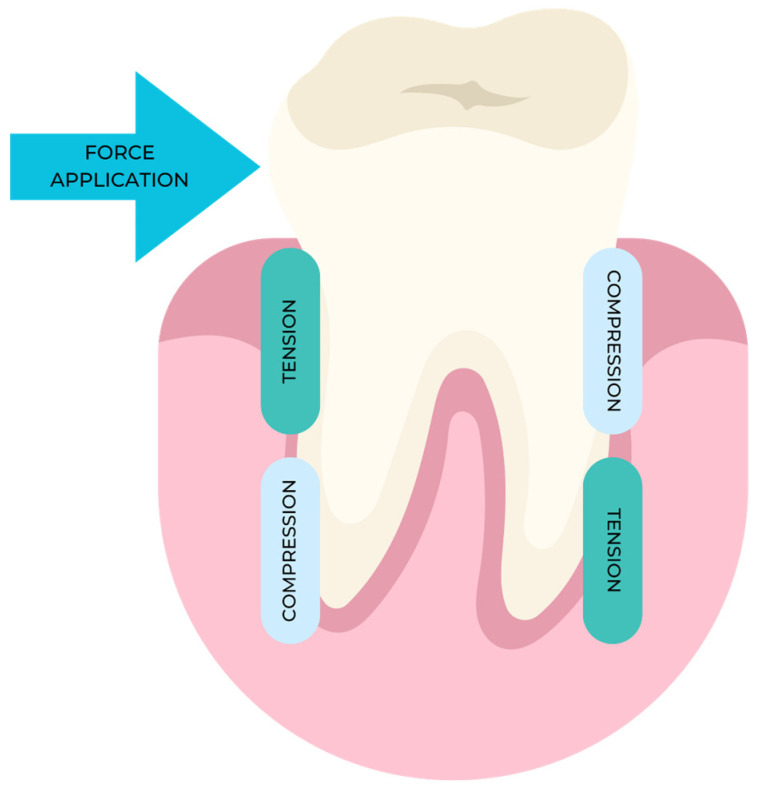
Compression and tension zones’ distributions based on the application of force.

According to the pressure–tension theory, chemical signals regulate cell development and, by extension, tooth movement, as shown in Figure 1.

When a constant strain is applied to a ligament, certain parts experience compression, decreasing oxygen tension and, eventually, blood flow, while other areas experience traction, increasing oxygen tension and, ultimately, blood flow [15]. Blood vessel damage leads to an inflammatory response, producing new blood vessels and connective tissue. When periodontal fibers are compressed, the “hyalinization” process [16] occurs, leading to the atrophy and/or pyknosis of cell nuclei and collagen fiber convergence in a gelatinous-like substance.

Hyalinization, as described by Reitan in his study of histological changes resulting from the orthodontic application of force [17], involves the loss of normal tissue architecture and discoloration characteristics of collagen present in processed histological material, occurring in cell-free regions inside the periodontal ligament. Reitan observed that hyalinization occurred within the periodontal ligament if even slight pressure was applied. Direct resorption occurs after removing hyalinized tissue due to the activated osteoclasts originating from the ligament, followed by indirect resorption with cellular materials from the blood flow. When the periodontal ligament (PDL) is compressed, blood flow within the PDL decreases until the blood vessels collapse completely, causing ischemia. After 1–2 s of gentle pressure, the PDL is partially compressed, causing fluids to leak out of both the periodontal space and the alveolus; after 3–5 s, blood vessels passively compress on the pressure side and dilate on the tension side, with the PDL’s fibers and cells appearing to be mechanically distorted [18,19,20].

These changes in blood flow and oxygen tension, as well as the release of prostaglandins and inflammatory cytokines, are triggered even by a brief application of pressure and its maintenance for a few minutes. Several chemical modulators are induced, cyclic adenosine monophosphate (cAMP) increases and cell differentiation occurs within the periodontal ligament after at least 4 h [21,22].

Orthodontic tooth movement initiation begins after 48 h, during which time the alveolar bone is remodeled due to the coordinated actions of the osteoclasts and osteoblasts. When the pressure exerted on the dental structure is high, the blood vessels in the periodontal ligament (PDL) collapse on the side experiencing compression. This collapse occurs within a 3−5 s time frame. Subsequently, after a few minutes, blood circulation in the PDL’s affected area is interrupted, resulting in sterile necrosis and the cellular components disappearing. These changes manifest as areas of hyalinization [22,23].

Therefore, the hyalinization and resorption processes indirectly lead to the necessary postponement of tooth displacement. This delay is accompanied by patient discomfort, primarily due to the ischemia and inflamed regions present within the periodontal ligament (PDL). The orthodontic forces’ optimal intensity occurs when they facilitate tooth movement by inducing cell differentiation, doing so while avoiding the complete occlusion of blood vessels in the periodontal ligament. Consequently, an orthodontic force’s biological impact is contingent upon either its intensity and the extent to which the periodontal ligament area is affected, or the pressure exerted on the tooth [24].

Thus, the orthodontic tooth movement process has three distinct phases. The initial phase is distinguished by prompt and swift tooth displacement, occurring within a 24 to 48 h time frame following the initial application of force. The observed rate is mostly influenced by tooth displacement within the periodontal ligament (PDL) space [24,25,26].

The lag phase, with a duration of 20 to 30 days, is characterized by minimal tooth displacement. The current stage is characterized by the PDL hyalinization process occurring in the compression area. No more tooth displacement is observed until the cells have fully eliminated the primary portion of the necrotic tissues. Following the lag phase, a subsequent period known as the post-lag phase occurs, where an increase in the pace of movement occurs [27].

**Figure 2:**
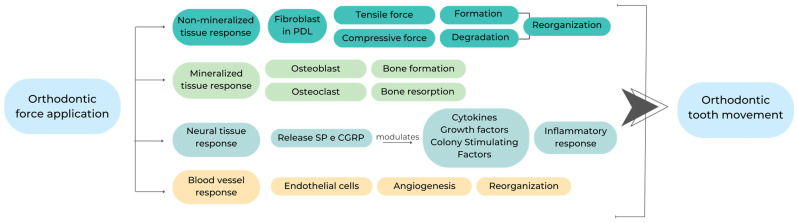
Effects of orthodontic force application on periodontal tissues.

The sequence of events following orthodontic tooth movement using appropriate biomarkers is described in Figure 2.

The interconnectedness of the periodontal tissues and tooth movement processes implies that orthodontic treatment is significant for addressing related issues. However, if tooth movement exceeds the alveolar process’ anatomical limits, it can worsen the periodontium’s destruction [28].

## 3. Periodontal and Bone Biomarkers Related to Orthodontic Force Application

Orthodontic tooth movement causes a cascade of coordinated cellular and molecular activities that result in connective tissue remodeling and osteoclast activation. During orthodontic tooth movement’s early stages, IL-1 is one of the most abundant cytokines in the periodontium [29].

IL-1 is primarily released by macrophages, with macrophage increases in compressed periodontal regions identified later during treatment. Thus, during tooth movement’s early stages, IL-1 is produced by other periodontal cell types, such as osteoclasts, as an immediate response to mechanical stress.

Previous studies have demonstrated the upregulation of inflammatory cytokines and their associated receptors following an inflammatory response generated via the perforation of the buccal cortical plate in rats undergoing orthodontic treatment. The mRNA concentrations of IL-1β in rats’ periodontal ligaments increase within a 3 h time frame following the application of an orthodontic force; this trend is primarily observed on the side experiencing pressure [30,31].

Therefore, applying IL-1RA therapy suppresses orthodontic tooth movement, as indicated by the decreased tooth displacement rate observed in IL-1RA-treated mice. Interleukin-1 beta (IL-1β) is widely acknowledged to be a potent inducer of interleukin-6 (IL-6) synthesis. It has functionalities comparable to those of IL-6 and tumor necrosis factor-alpha (TNF-α) [32].

Tumor necrosis factor-α (TNF-α) is a pro-inflammatory cytokine demonstrated to induce both acute and chronic inflammation, as well as promote bone resorption [33].

Additional clinical and animal studies have also established the primary involvement of prostaglandins E (namely PGE1 and PGE2) in promoting bone loss [34,35]. Prostaglandins are synthesized from arachidonic acid, originating from phospholipids. The initial reaction to pressure stimulation releases prostaglandins. This release occurs when cells undergo mechanical deformation, mobilizing membrane phospholipids. As a result, inositol phosphate (IP), a significant chemical messenger, is generated. Furthermore, the PGE2 level is an indicator of the biological processes that occur in the periodontium during orthodontic tooth movement. A prior study observed that a large increase in PGE2 levels occurs on both the tension and compression sides [36].

During the orthodontic movement process, several proliferative markers are expressed. The presence of KI-67 and the receptor activator of nuclear factor-Kappa β ligand (RANKL) [37,38] suggests the recruitment of osteoclasts in regions experiencing compression. Conversely, the expression of Runx2 [39], Col1-GFP, and BSP-GFP in cells indicates increases in differentiated osteoblasts in regions experiencing tension [40].

In the early stages of orthodontic tooth movement, the macrophage colony-stimulating factor (M-CSF) is key to osteoclast differentiation, increasing osteoclast recruitment and differentiation rates [41]. In particular, optimal M-CSF doses are associated with observable changes in tooth movement and gene expression. This relationship will enable future clinical studies for accelerating tooth movement methods.

Vascular endothelial growth factor (VEGF) is the principal mediator of angiogenesis, and it increases vascular permeability during tissue neoformation, usually due to the presence of blood vessels [42]. Periodontal ligament angiogenesis and vascular endothelial growth factor activation [43] are both triggered by compressive forces used in orthodontic tooth movement.

When teeth are moved for orthodontic treatment, neurogenic inflammation manifests as an increase in certain proteins’ concentrations in the periodontium. Periodontal peripheral nerve fibers transmit impulses to the central nervous system via somatosensory neurons. When orthodontic tension is physiologically applied to the gums, the nerve gums’ fibers release the neurotransmitters calcitonin gene-related peptide (CGRP) and substance P [44].

Orthodontic tooth movement causes a biological change, primarily in the bone tissue around the teeth. Osteoblasts and osteoclasts express alkaline phosphatase (ALP) and acid phosphatase (ACP), respectively, which are both involved in bone metabolism [45].

The periodontal ligament has significantly higher ALP activity than other connective tissues [46]. These enzymes are created in the periodontium in response to orthodontic force, and they diffuse in the gingival crevicular fluid at the affected location. Orthodontic tooth movement-related tissue changes can be observed by monitoring phosphatase activity in the gingival crevicular fluid. Prior human and animal studies have linked changes in GCF phosphatase activity to alveolar bone remodeling [47,48,49].

To identify histological and biochemical changes in bone turnover and, by extension, the rate/amount of tooth movement, we must analyze the ALP associated with bone metabolism under healthy gingival settings. Further study of this enzyme’s diagnostic potential in orthodontics is warranted because it can differentiate between teeth being clinically relocated and unmoved teeth [50].

## 4. Orthodontic Fixed Appliance

For correcting various malocclusions, fixed orthodontic treatment remains the gold standard [29,30,31,32,33,34,35,36,37,38,39,40,41,42,43,44,45,46,47,48,49,50,51]. Though traditional braces have been widely acknowledged as useful, there are still drawbacks associated with this treatment option. Plaque buildup in the teeth is encouraged by the use of a fixed orthodontic appliance because it makes cleaning teeth more difficult [52,53,54]. Several studies have analyzed the correlation between bracket materials, designs, and ligations and the prevalence of cariogenic bacteria and periodontal damage [55,56,57]. For bracket ligation techniques, it has previously been hypothesized that self-ligating brackets (SLBs) exhibit superior periodontal outcomes due to the absence of ligature materials and reduced number of sites promoting retention [58].

### 4.1. Self-Ligating Brackets (SLBs) and Conventional Brackets (CBs)

The existing literature contains only a limited number of studies with varying outcomes for comparing self-ligating brackets (SLBs) to conventional brackets (CBs) for elastomeric ligatures [58]. Several studies have documented that using elastomeric ligated ceramic brackets (CBs) leads to increased plaque accumulation and periodontal inflammation compared to self-ligating brackets (SLBs) [3,59]. However, conflicting findings have also been reported, with other studies indicating no statistically significant difference between the two bracket types for either factor [56]. In contrast, previous studies also documented increased bacterial colonization and compromised periodontal health resulting from the use of subgingival lingual brackets (SGLBs) [60]. Although SGLBs prevent the utilization of ligatures, they nonetheless have opening and closing mechanisms that have the potential to create extra plaque retention areas. White demineralization, caries, or periodontal disease areas can form at numerous plaque retention sites [61,62,63].

Multiple studies have shown patients undergoing orthodontic treatment [64,65,66] to have an increased level of cariogenic bacteria (such as Streptococcus mutans and Lactobacillus sp.) and, possibly, pathogenic Gram-negative bacteria.

Pellegrini et al. [57] and Mummola et al. [55] reported that using self-ligating brackets (SLBs) reduced bacterial levels compared to those recorded when using elastomeric ligating brackets. In contrast, van Gastel et al. [58] observed greater bacterial colonization due to SLB use. In line with this observation, Nalçaçı et al. [64] and Pandis et al. [53] also found no significant disparity in salivary SM levels when comparing elastomeric ligated conventional brackets (CBs) and self-ligating brackets (SLBs).

In their study, Carillo et al. [65] observed that there were no statistically significant variations in SM and LB levels during the one-month treatment period. Nevertheless, they observed a deterioration in periodontal health. Another study revealed that one week after bonding, individuals with SLBs had greater levels of anaerobic and aerobic bacteria colonization, as well as enhanced gingival hypertrophy, compared with those with CB. However, no significant disparity in bleeding via probing was identified between the two cohorts [58].

### 4.2. Changes in Oral Microbiota

*Candida* is a commensal, benign bacterium found in the oral cavity of 53% of people worldwide; however, if aberrations in the microbiota’s usual range or host immunological deficiency exist, this bacterium may grow to aggressive and dangerous levels [64]. The colonization of candida and the prevalence of specific strains or species exhibit temporal variability during orthodontic therapy. Contaldo et al.’s [62] review underscored the lack of clarity regarding the presence of *Candida* sp., viruses and protozoa in orthodontic patients’ oral microbiota.

*Candida* spp. was more prevalent 6 months after bonding orthodontic appliances compared with the pre-bonding period, while *C. tropicalis* was identified in 20% of patients in Hernández-Solise et al.’s study [67]. Furthermore, the studies of Perkowski et al. [68] and Grzegocka et al. [69] both found that orthodontic treatment positively impacts the colonization of *Candida* sp. This correlation has specifically been observed in cases in which fixed appliances were utilized, while the extent of colonization might vary throughout the duration of treatment.

Guo et al. [70] conducted a comprehensive review and meta-analysis encompassing 13 articles. Their research objective was to examine microbiological alterations in subgingival plaque among individuals undergoing orthodontic treatment. They found that the presence of subgingival infections increases during orthodontic therapy, although only briefly in most cases.

Bergamo et al. noted a decline in *Candida* sp. levels 60 days following bracket bonding. However, they only noted a specific decrease in the representation of *C. albicans* after a 90-day period [71].

Notwithstanding the globally employed conventional procedures’ acknowledged efficacy, we must acknowledge the inherent limitations of these approaches. For example, traditional dental techniques are inconvenient and can potentially induce pain, frequently making oral hygiene maintenance more challenging. The main components of fixed orthodontic appliances, namely brackets, bands, ligatures, and orthodontic wires, may impede the natural self-cleaning mechanisms of the tongue and cheeks. Additionally, these components may increase bacterial plaque accumulation and alter both the qualitative and quantitative aspects of the bacterial population [72].

Fixed appliances’ impacts on oral microbiota’s composition and characteristics is a transient phenomenon contingent on controlling oral hygiene. Patients are advised to use caution when handling the stent and frequently remove the plaque accumulating around the wire to enhance the oxidation-reduction potential. Hence, using an alternative removable orthodontic appliance should make this process easier to perform and promote improved recovery in individuals in need of prompt interventions [72,73].

### 4.3. Changes in Periodontal Health

Debate about the association between gingival recessions and orthodontic therapy is ongoing. The prevalence of this relationship ranges from 5% to 12% upon the completion of treatment, but it may rise to 47% during the course of long-term surveillance [74]. Certain writers have proposed that orthodontic treatment is linked to the occurrence of alveolar bone loss, gingival recession, and reduction in the clinical attachment level [74].

Numerous inquiries have been conducted into the identities of individuals receiving medical treatment using both permanent and removable apparatus in recent times, as shown in Table 1. The conclusion derived from these papers, meanwhile, is a subject of ongoing academic discussion.

Orthodontic treatment is associated with a high prevalence of periodontal problems [6]. Issues commonly observed through the use of orthodontic fixed appliances include gingivitis, periodontitis, gingival recession or hypertrophy, and alveolar bone loss.

## 5. Orthodontic Removable Appliance and Periodontal Health

Multiple studies have demonstrated that using orthodontic fixed appliances (FA) leads to a notable increase in dental plaque accumulation. This trend is primarily attributed to the challenges related to maintaining adequate oral hygiene while wearing such appliances. Consequently, this heightened plaque accumulation causes gradual enamel demineralization and gingival inflammation. Ultimately, these factors may deteriorate the tissues supporting the teeth [80].

Transparent plastic clear aligners (CAs) have been used to address some restrictions associated with traditional fixed appliances (FAs). The existing literature characterizes this treatment option as a secure, comfortable, and visually pleasing intervention [81].

In Pango Madariaga et al.’s study [76], the authors observed that multibracket appliances exhibited considerable GBI increases compared to aligners during the initial evaluation. However, they also found that the appliance type did not discernibly impact periodontal variable enhancement. This finding remained consistent for all factors, such as aging, and all sites assessed, even if these factors were statistically significant. Thus, the authors emphasized the need to consider alternative criteria to assess an appliance’s efficacy. Similarly, Chhibber et al. [80] presented findings that challenge the prevailing notion that removable appliances, relative to multibracket ones, have few adverse periodontal health impacts.

Levrini et al. [81] reported a statistically significant distinction between the removable aligner and fixed appliance groups in terms of the plaque index (PLI), gingival bleeding index (GBI), and probing depth (PD), with patients using aligners having the lowest average values. The researchers found that removable appliances should be the primary therapy option for individuals susceptible to periodontal disease.

Similarly, Abbate et al. performed a microbiological examination, although no patients exhibited periodontopathic anaerobes following a 12-month therapy period. During the first period of up to one year of therapy, the full mouth plaque (FMPS) value experienced a threefold increase, while the full mouth bleeding (FMBS) value doubled among adolescents undergoing treatment with multibracket appliances. Conversely, both the FMPS and FMBS scores decreased in adolescents using aligners [82].

Azaripour et al. [83] reported an increase in dental plaque accumulation for users of both orthodontic appliances, with a greater increase observed in the multibracket group than in the aligners group.

Miethke and Vogt conducted a clinical trial in which all the indexes improved between the initial and the final screening stages, regardless of the orthodontic appliance used [84].

At the outset, there were no statistically significant disparities detected in the GI and PBI variables. Furthermore, the results indicated a statistically significant decrease in the periodontal index (PI) among individuals who underwent treatment with aligners. At the outset, no statistically significant disparities were identified in the GI and PBI variables. Furthermore, the data indicated a statistically significant decrease in periodontal inflammation (PI) among individuals who underwent treatment with aligners. The study’s authors concluded that no discernible disparities existed between the initial and ongoing treatment outcomes for multibracket appliances and aligners. They attributed enhanced dental hygiene to many factors, alongside other criteria.

Issa et al. [85] conducted a study revealing a notable disparity in plaque levels between patients treated with aligners and those undergoing traditional treatment. The aligner-treated patients had significantly lower levels of plaque compared with their counterparts. Furthermore, the authors showed that individuals who underwent aligner treatment exhibited superior scores for all seven recorded measures.

Numerous physicians hold the belief that CAs are better able to preserve periodontal health than conventional FAs. However, the available literature is insufficient to substantiate this theory [86,87].

## 6. Discussion and Perspectives

Significant macroscopic and microscopic alterations were observed in the alveolar bone and periodontal ligaments subjected to varying degrees of amplitude, frequency, and duration of forces such as pressure. Applying stress on the tooth also causes changes in the periodontal tissue’s circulation and blood flow, leading to the production and release of different molecules, including cytokines, growth factors, colony-stimulating factors, enzymes, and neurotransmitters, at the local level.

Orthodontic therapy should be used to straighten the teeth while causing minimal gum and root damage. One negative side effect of orthodontic treatment is root resorption [88]. Root resorption has been studied for 150 years.

The process of using cementoclastic or osteoclastic methods to break down dentin or cement is called root resorption [89]. This process can shorten or blunt the root. Root resorption can also be described as small patches of resorption lacunae observed using histology methods [90]. Apical root resorption is likely impacted by mechanical factors, genetic propensity, and individual biological variability [91,92].

Identifying various biomarkers that indicate biological changes after applying orthodontic forces is highly valuable for selecting an optimal mechanical force to achieve the desired tooth movement rate and expedite orthodontic treatment. This approach mitigates potential negative consequences such as root resorption or bone loss. Apical root resorption is caused by a mixture of mechanical causes, individual biological variability levels, and orthodontic forces [92]. In Lupi et al.’s study, root resorption was 15% before orthodontic treatment and 73% after it [93].

As permanent appliances allow for a greater range of tooth movement, they more effectively induce root resorption than removable appliances [94]. Research into the potential for root resorption due to various bracket designs has produced conflicting findings [95,96]. A statistical analysis of root resorption results from three trials comparing self-ligating devices with traditional edgewise methods found no significant differences [97,98,99].

In general, having a comprehensive understanding of the dynamic processes taking place in periodontal tissues during orthodontic interventions can facilitate the selection of appropriate mechanical loading techniques. Human premolar teeth with 50 g, 100 g, and 200 g of intrusive force were examined by Harry and Sims using a scanning electron microscope. They concluded that larger forces accelerated root resorption by increasing root surface stress, in turning accelerating the development of lacunae [100].

Although Owman-Moll et al. discovered a high degree of interindividual variability in root resorption frequency and severity, they did not identify any statistically significant differences. They found that the force’s magnitude had no effect on root resorption, but the impact of individual reactions may have been greater [101].

The question of whether greater root resorption occurs with continuous or intermittent pressures is still up for debate. As the tooth cannot move with a discontinuous force applied, the resorbed cementum has time to repair, leading to the assumption that this type of force causes less root resorption [102,103]. In contrast, Owman-Moll et al. [101] observed that applying a buccally directed force of 50 g to human premolars, either continuously or intermittently, did not affect root resorption’s degree or severity.

This approach, in turn, can reduce the treatment duration and mitigate potential negative outcomes associated with orthodontic treatment.

The researchers Sameshima and Sinclair found that the degree of root resorption is proportional to the root’s migration distance. One reason that maxillary incisors are more likely to experience root resorption is that they are moved more frequently during orthodontic treatment compared with other teeth [104,105].

The correlation between orthodontic interventions and the state of periodontal tissues is a complex issue, particularly regarding periodontal well-being during and following orthodontic therapy. As orthodontic treatment during inflammation causes the periodontium to rapidly and irreversibly break down, periodontal therapy is typically completed before orthodontic treatment [106].

In past studies, it is widely accepted that the appropriate application of orthodontic appliances does not detrimentally affect the periodontium in its proximity. Orthodontic appliances also provide support for periodontal tightness and splinting in cases where oral hygiene is maintained.

Numerous clinical investigations have documented increases in plaque buildup and gingivitis occurrence in individuals undergoing orthodontic treatment. Furthermore, orthodontic treatment has induced changes in oral bacteria’s composition and diversity. Three months after orthodontic treatment began, a study comparing the oral microbiota of patients receiving orthodontic treatment to those of subjects not receiving orthodontic therapy revealed a statistically significant increase in microbial counts for various periodontopathic bacteria, including *P. gingivalis* [107].

An intriguing study conducted by Naranjio examined the relationship between changes in clinical parameters and subgingival plaque in subgingival microbial sample cultures taken from orthodontic patients before and after bracket placement, as well as from control subjects who were not receiving orthodontic treatment. The results showed that the levels of *P. gingivalis*, *P. media*/*Prevotella nigrescens*, *T. forsythia*, and *Fusobacterium* spp. were higher in patients three months after orthodontic therapy than in both the control and baseline groups [108].

Through their modulation of osteoclast development and matrix metalloproteinase production, pro-inflammatory cytokines such as tumor necrosis factor-α (TNF-α), interleukin-1 (IL-1), IL-6, and IL-8 enable orthodontic tooth movement, according to multiple studies [109,110]. Recent animal-based research revealed that orthodontic tooth movement exacerbates periodontitis by increasing IL-1 and TNF- levels [111].

Variables influencing the synthesis and/or activity of these cytokines regulate the orthodontic tooth movement rate. Orthodontic tooth movement is slowed when soluble receptors to IL-1 or TNF-α or neutralizing antibodies to VEFG are administered locally. On the other hand, the tooth movement rate increases when the expression of pro-inflammatory cytokines near the moving teeth is stimulated via osteo-perforation.

In addition, other studies have shown that the antioxidant n acetyl cysteine (NAC) reduces pro-inflammatory cytokine generation in gingival fibroblasts when exposed to lipopolysaccharide, while NAC reduces alveolar bone loss in experimental periodontitis [112,113].

Recently, we have witnessed significant progress in the development and production of dental motion materials through computer-aided design and manufacturing. This shift has increased interest in using enhanced specifications for orthodontic treatment technologies [114].

Nevertheless, numerous studies have showcased current appliances’ ability to rectify and manage various illnesses, including both minor and severe malocclusion, while improving periodontal health [115].

Extensive research has been conducted into the orthodontic therapy–periodontitis relationship, encouraging ongoing discussions about the impact of orthodontic treatment on periodontitis’ prevalence [116].

The results should be interpreted with caution because there are some limitations to this study’s methodology. Firstly, the cause-and-effect relationship between orthodontic treatment and the reduction in periodontitis’ prevalence is a controversial subject; therefore, it is hard to generalize these results on a global scale.

To better understand the orthodontic therapy–periodontitis reduction relationship, better-controlled, long-term studies are necessary.

## 7. Conclusions

When using orthodontics to treat individuals with periodontal problems, we must pay attention to the following aspects: the orthodontic appliance utilized, the state of the patient’s teeth and periodontal tissues, the depth of the gum pockets, the position of the teeth in the supporting tissue, and the patient’s motivation and ability to sustain oral hygiene and health. Orthodontic therapy’s success depends on an orthodontist’s ability to anticipate and address any periodontal issues arising before, during, or after treatment. However, process conduction may overcome periodontal defects under specific conditions.

## Figures and Tables

**Table 1:** Outcomes of studies of fixed and removable appliances.

Authors	Year of Publication	Parameters	Outcome Measures
Liu et al. [75] (clinical study)	2011	PLI, GI, PD	PLI, GI, and PD increased significantly in the first 3 months following appliance insertion but decreased significantly in the first 6 months after appliance removal.
Madariaga et al. [76] (prospective clinical study)	2020	PLI, GI, GBI, PD	According to the linear regression models, appliance type did not affect periodontal variables. Thus, fixed appliance and clear aligner orthodontic patients had similar gingival health statuses.
Wu Y. et al. [77] (meta-analysis)	2020	PLI, GI, PD	Removable appliances had significantly lower PLIs than fixed equipment. At 6 months, removable appliances reduced GI, but they did not do so at 3 months. At 3 months, mobile devices had lower PDs than fixed appliances.
Li W. et al. [78] (clinical study)	2017	PLI, GI, PD, GBI	After 3 months, Invisalign therapy lowered GBI and PD levels compared to fixed treatment (*p* < 0.05); however, there was no significant difference after 6 months (*p* > 0.05).
Eroglu et al. [27] (clinical study)	2019	PLI, GI, PD, GBI	The authors found no significant differences in the plaque index, gingival index, bleeding on probing, or probing depth (*p* > 0.05).
Sun et al. [79] (prospective clinical study)	2018	PLI, GI, PD, GBI	Before and three months after treatment, the two patient groups had similar periodontal indicators.

GI = gingival index; PLI = plaque index; PD = probing depth; GBI = gingival bleeding index.

## Data Availability

All data are available from the corresponding authors upon reasonable request.
